# Centered Kernel Alignment Enhancing Neural Network Pretraining for MRI-Based Dementia Diagnosis

**DOI:** 10.1155/2016/9523849

**Published:** 2016-04-11

**Authors:** David Cárdenas-Peña, Diego Collazos-Huertas, German Castellanos-Dominguez

**Affiliations:** Signal Processing and Recognition Group, Universidad Nacional de Colombia, Manizales, Colombia

## Abstract

Dementia is a growing problem that affects elderly people worldwide. More accurate evaluation of dementia diagnosis can help during the medical examination. Several methods for computer-aided dementia diagnosis have been proposed using resonance imaging scans to discriminate between patients with Alzheimer's disease (AD) or mild cognitive impairment (MCI) and healthy controls (NC). Nonetheless, the computer-aided diagnosis is especially challenging because of the heterogeneous and intermediate nature of MCI. We address the automated dementia diagnosis by introducing a novel supervised pretraining approach that takes advantage of the artificial neural network (ANN) for complex classification tasks. The proposal initializes an ANN based on linear projections to achieve more discriminating spaces. Such projections are estimated by maximizing the centered kernel alignment criterion that assesses the affinity between the resonance imaging data kernel matrix and the label target matrix. As a result, the performed linear embedding allows accounting for features that contribute the most to the MCI class discrimination. We compare the supervised pretraining approach to two unsupervised initialization methods (autoencoders and Principal Component Analysis) and against the best four performing classification methods of the 2014* CADDementia* challenge. As a result, our proposal outperforms all the baselines (7% of classification accuracy and area under the receiver-operating-characteristic curve) at the time it reduces the class biasing.

## 1. Introduction

In 2010, the number of people aged over 60 years with dementia was estimated at 35.6 million worldwide and this figure had been expected to double over the next two decades [1]. Actually, World Health Organization and the Alzheimer's Disease International had declared dementia as a public health priority, encouraging articulating government policies and promoting actions at international and national levels [2]. Alzheimer's disease (AD) is the most diagnosed dementia-related chronic illness that demands very expensive costs of care, living arrangements, and therapies. Thus, efforts are underway to improve treatment which may delay, at least, one year the AD onset and development, leading to decreasing the number of cases by nine millions [3]. AD can be early diagnosed by predicting the conversion to dementia from a state of mild cognitive impairment (MCI) that especially increases the AD risk [4].

In this regard, early diagnosis is directly related to the effectiveness of interventions [5]. Along with clinical history, neuropsychological tests, and laboratory assessment, the joint clinical diagnosis of AD also includes neuroimaging techniques like positron emission tomography (PET) and magnetic resonance imaging (MRI). These techniques are usually incorporated in the routine workup for excluding secondary pathology causes (e.g., tumors) [6, 7]. However, factors related to image quality and radiologist experience may limit their use [8]. For dealing with this issue, the imaging-based automatic assessment of quantitative biomarkers has been proven to enhance the performance for dementia diagnosis. In the particular case of AD, there are two groups of widely studied biomarkers: (i) patterns of brain amyloid-beta, such as low cerebrospinal fluid (CSF) *Aβ*42 and amyloid PET imaging, and (ii) measures of neuronal injury or degeneration like CSF tau measurement, fluorodeoxyglucose PET, and atrophy on structural MRI [9]. Thus, structural MRI has become valuable for biomarker assessment since this noninvasive technique explains structural changes at the onset of cognitive impairment [10].

For the purpose of automated diagnosis, the first stage to implement is the structure-wise feature extraction from available MRI data, including voxel-based morphometry, volume, thickness, shape, and intensity relation. Nonetheless, more emphasis usually focuses on the classification approach due to its strong influence on the entire diagnosis system. With regard to neurodegenerative diseases, the reported classifiers range from straightforward approaches (*k*-Nearest Neighbors [11], Linear Discriminant Analysis [12], Support Vector Machines [13], Random Forests [14], and Regressions [15]) to the combination of classifiers [16]. Most of the above approaches had been evaluated for the* 2014 CADDementia challenge* which aimed to reproduce the clinical diagnosis of 354 subjects in a multiclass classification problem of three diagnostic groups [17], Alzheimer's diagnosed patients, subjects with MCI, and healthy controls (NC), given their T1-weighted MRI scans. As a result, the best-performing algorithm yielded an accuracy of 63.0% and an area under the receiver-operating-characteristic (ROC) curve of 78.8%. Nonetheless, reported true positive rates are 96.9% and 28.7% for NC and MCI, respectively, resulting in class biasing.

Generally speaking, dementia diagnosis from MRI still remains a challenging task, mainly, because of the nature of mild cognitive impairment; that is, there is a heterogeneous and intermediate category between the NC and AD diagnostic groups, from which subjects may convert to AD or return to the normal cognition [4]. For overcoming this shortcoming, machine learning tools as the artificial neural networks (ANN) have been developed to enhance dementia diagnosis, presenting the following advantages [18, 19]: (i) ability to process a large amount of data, (ii) reduced likelihood of overlooking relevant information, and (iii) reduction of diagnosis time.

Nonetheless, an essential procedure for ANN implementation is initializing deep architecture (termed pretraining) which can be carried out by training a deep network to optimize directly only the supervised objective of interest, starting from a set of randomly initialized parameters. However, this strategy performs poorly in practice [20]. With the aim to improve each initial-random guess, a local unsupervised criterion is considered to pretrain each layer stepwise, trying to produce a useful higher-level description based on the adjacent low-level representation output of the previous layer. Particular examples that use unsupervised learning are the following: Restricted Boltzmann Machines [21], autoencoders [22], sparse autoencoders [23], and the greedy layer-wise unsupervised learning which is the most common approach that learns one layer of a deep architecture at a time [24]. Although the unsupervised pretraining generates hidden representations that are more useful than the input space, many of the resulting features may be irrelevant for the discrimination task [25, 26].

In this paper, we benefit from the ANN advantages for complex classification tasks to introduce a novel supervised ANN initialization approach devoted to the automated dementia diagnosis. The proposed pretraining approach searches for a linear projection into a more discriminating space so that the resulting embedding features and labels become as much as possible associated. Consequently, the obtained ANN architecture should match better the nature of supervised training data. Taking into account the fact that the ANN straightforward hybridization with other approaches yields stronger paradigms for solving complex and computationally expensive problems [27, 28], we also incorporate kernel theory for assessing the affinity between projected data and available labels. The use of kernel approaches offers an elegant, functional analysis framework for tasks, gathering multiple information sources (e.g., features and labels) as the minimum variance unbiased estimation of regression coefficients and least squares estimation of random variables [29]. Moreover, we consider the centered kernel alignment criterion as the affinity measure between a data kernel matrix and a target label matrix [30, 31]. As a result, the linear embedding allows accounting for features that contribute the most to the class discrimination.

The present paper is organized as follows: Section 2 firstly describes the mathematical background on learning projections using CKA and ANN for classification.  Section 3 introduces all the carried out experiments for tuning the algorithm parameters and the evaluation scheme with blinded data. Then, achieved results are discussed in Section 4. Finally, Section 5 presents the concluding remarks and future research directions.

## 2. Materials and Methods

### 2.1. Classification Using Artificial Neural Networks

Within the classification framework, an *L*-layered ANN is assumed to predict the needed class label set through a battery of feedforward deterministic transformations, which are implemented by the hidden layers **h** ^*l*^, which map the input space **x** to the network output **h** ^*L*^ as follows [27]:

(1) hl=ϕbl+Wlhl−1,∀l=1,…,L−1,h0=x,

where **b** ^*l*^ ∈ *ℝ* ^*m*_*l*+1_^ is the *l*th offset vector, **W** ^*l*^ ∈ *ℝ* ^*m*_*l*+1_×*m*_*l*_^ is the *l*th linear projection, and *m*_*l*_ ∈ *ℤ* ^+^ is the size of the *l*th layer. The function *ϕ*(·) ∈ *ℝ* applies saturating, nonlinear, element-wise operations. Here, we choose the standard sigmoid, *ϕ*(*z*) = sigmoid(*z*), expressed as follows:

(2) $$\begin{array}{c} \text{sigmoid}\left(\;z\;\right)=\frac{\left(\tanh\left(z\right)+1\right)}{2}. \end{array}$$

The first layer in (1) (i.e., **h** ^0^ ∈ *ℝ* ^*D*^) is conventionally adjusted to the input feature vector. In turn, the output layer **h** ^*L*^ ∈ [0,1]^*C*^ predicts the class when combined with a provided target *t* ∈ {1,…, *C*} into a loss function *ℒ*(**h** ^*L*^, *t*). In practice, the output layer can be carried out by the nonlinear softmax function described as follows:

(3) $$\begin{array}{c} h_{c}^{L}=\frac{\exp\left(b_{c}^{L}+w_{c}^{L}h^{L-1}\right)}{\sum_{j}\exp\left(b_{c}^{L}+w_{c}^{L}h^{L-1}\right)}, \end{array}$$

where *b*_*c*_^*L*^ is the *c*th element of **b** ^*L*^, **w**_*c*_^*L*^ is the *c*th row of **W** ^*L*^, **h** ^*L*^ is positive, and ∑_*c*_ *h*_*c*_^*L*^ = 1.

The rationale behind the choice of softmax function is that each yielded output *h*_*c*_^*L*^ can be used as an estimator of *P*(*t*_*i*_ = *c*∣**x**_*i*_), so that the interpretation of *t*_*i*_ relates to the class associated with input pattern **x**_*i*_. In this case, the softmax loss function corresponds often to the negative conditional log-likelihood:

(4) $$\begin{array}{c} L\left(\;h^{L},t\;\right)=-log\underset{c}{\sum }P\left(\;t=c\;|\;x\;\right). \end{array}$$

Therefore, the expected value over (**x**, *t*) pairs is minimized with respect to the biases and weighting matrices.

### 2.2. ANN Pretraining Using Centered Kernel Alignment

Let **X** ∈ {**x**_*i*_ ∈ *ℝ* ^*D*^ : *i* ∈ *N*} be the input feature matrix with size *ℝ* ^*D*×*N*^ which holds *N* trajectories and let **x**_*i*_ ⊂ *𝒳* be a *D*-dimensional random process. In order to encode the affinity between a couple of trajectories, {**x**_*i*_, **x**_*j*_}, we determine the following kernel function:

(5) $$\begin{array}{c} \kappa \left(\;x_{i},x_{j}\;\right)=\langle \;\varphi \left(\;x_{i}\;\right),\varphi \left(\;x_{j}\;\right)\;\rangle ,\;\forall i,j\in N. \end{array}$$

〈·, ·〉 stands for the inner product and *φ*(·) : *ℝ* ^*D*^ → *ℋ* maps from the original domain, *ℝ* ^*D*^, into a Reproduced Kernel Hilbert Space (RKHS), *ℋ*. As a rule, it holds that |*ℋ* | →*∞*, so that |*ℝ* ^*D*^ | ≪|*ℋ*| can be assumed. Nevertheless, there is no need for computing *φ*(·) directly. Instead, the well-known* kernel trick* is employed for computing (5) through the positive definite and infinitely divisible kernel function as follows:

(6) $$\begin{array}{c} k_{ij}=\kappa \left(\;d\left(\;x_{i},x_{j}\;\right)\;\right), \end{array}$$

where *d* : *ℝ* ^*D*^ × *ℝ* ^*D*^ ↦ *ℝ* ^+^ is a distance operator implementing the positive definite kernel function *κ*(·). A kernel matrix **K** ∈ *ℝ* ^*N*×*N*^ that results from the application of *κ* over each sample pair in **X** is assumed as the covariance estimator of the random process *𝒳* over the RKHS.

With the purpose of improving the system performance in terms of learning speed and classification accuracy, we introduce the prior label knowledge into the initialization process. Thus, we compute the pairwise relations between the feature vectors through the introduced feature similarity kernel matrix **K** ∈ *ℝ* ^*N*×*N*^ which has elements as follows:

(7) $$\begin{array}{c} k_{ij}=\kappa_{x}\left(\;d_{W}\left(\;x_{i},x_{j}\;\right)\;\right),\;\forall i,j\in \left\{1,\ldots ,N\right\}, \end{array}$$

with *d*_*W*_ : *ℝ* ^*D*^ × *ℝ* ^*D*^ ↦ *ℝ* ^+^ being a distance operator that implements the positive definite kernel function *κ*_**x**_(·), and {(**x**_*i*_, *t*_*i*_):  *i* = 1,…, *N*} is a set of input-label pairs with **x**_*i*_ ∈ *ℝ* ^*D*^ and *t*_*i*_ ∈ {1, *C*}, with *C* being the number of classes to identify.

Since we look for a suitable weighting matrix for initializing the ANN optimization, we rely on the Mahalanobis distance that is defined on a *D*-dimensional space by the following inverse covariance matrix **W** ^*⊤*^ **W**:

(8) $$\begin{array}{c} d_{W}\left(\;x_{i},x_{j}\;\right)={\left(\;x_{i}-x_{j}\;\right)}^{⊤}W^{⊤}W\left(\;x_{i}-x_{j}\;\right), \end{array}$$

where matrix **W** ∈ *ℝ* ^*m*_1_×*D*^ holds the linear projection **y**_*i*_ = **W** **x**_*i*_, with **y**_*i*_ ∈ *ℝ* ^*m*_1_^, *m*_1_ ≤ *D*.

Based on the already estimated feature similarities, we propose further to learn the matrix **W** by adding the prior knowledge about the feasible sample membership (e.g., healthy or diseased groups) enclosed in a matrix **B** ∈ *ℝ* ^*N*×*N*^ with elements *b*_*ij*_ = *δ*(*t*_*i*_ − *t*_*j*_). Thus, we measure the similarity between the matrices **K** and **B** through the following function of centered kernel alignment (CKA) [32]:

(9) $$\begin{array}{c} \rho \left(\;K,B\;\right)=\frac{{\langle HKH,HBH\rangle }_{F}}{{\left\|HKH\right\|}_{F}{\left\|HBH\right\|}_{F}},\;\rho \in \left[0,1\right], \end{array}$$

where **H** = **I** − *N* ^−1^11^*⊤*^, with **H** ∈ *ℝ* ^*N*×*N*^, is a centering matrix, 1 ∈ *ℝ* ^*N*^ is an all-ones vector, and 〈·, ·〉_*F*_ and ‖·,·‖_*F*_ stand for the Frobenius inner product and norm, respectively.

Therefore, the centered version of the alignment coefficient leads to better correlation estimation compared to its uncentered version [31]. Therefore, the CKA cost function, described in (9), highlights relevant features by learning the matrix **W** that best matches all relations between the resulting feature vectors and provided target classes. Consequently, we state the following optimization problem to compute the projection matrix:

(10) $$\begin{array}{c} W^{⋆}=\underset{W}{\arg\;\max}\;\rho \left(\;K_{W},B\;\right), \end{array}$$

and we thus initialize the first layer of the ANN with **W** ^⋆^.

Additionally, the weighting matrix allows analyzing the contribution of the input feature set for building the projection matrix by computing the feature relevance vector **ϱ** ∈ *ℝ* ^*D*^ in the following form:

(11) $$\begin{array}{c} \varrho_{d}=E\left\{\;w_{ud}^{2}:\forall u\in \left[\;1,m_{1}\;\right]\;\right\}, \end{array}$$

where *w*_*ud*_ ∈ *ℝ* is the weight that associates each *d*th feature to *u*th hidden neuron. *E*{·} stands for the averaging operator. The main assumption behind the introduced relevance in (11) is that the larger the values of *ϱ*_*d*_ the larger the dependency of the estimated embedding on the input attribute.

## 3. Experimental Setup

**Figure 1:**
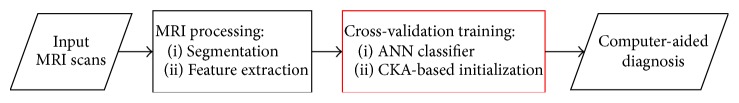
General processing pipeline: FreeSurfer independently segments and extracts features from given MRIs. Centered kernel alignment is proposed to learn a projection matrix initializing the NN training in a 5-fold cross-validation scheme. Tuned model is used for classification task.

An automated, computer-aided diagnosis system based on artificial neural networks is introduced to classify structural magnetic resonance imaging (MRI) scans in accordance with the following three neurological classes: normal control (NC), mild cognitive impairment (MCI), and Alzheimer's disease (AD).  Figure 1 illustrates the methodological development of the proposed approach.

### 3.1. ADNI Data

Data used in the preparation of this paper were obtained from the Alzheimer's Disease Neuroimaging Initiative (ADNI) database (http://adni.loni.usc.edu/) which was launched in 2003 by the National Institute on Aging (NIA), the National Institute of Biomedical Imaging and Bioengineering (NIBIB), the Food and Drug Administration (FDA), private pharmaceutical companies, and nonprofit organizations. The primary goal of ADNI is to test whether serial magnetic resonance imaging (MRI), positron emission tomography (PET), other biological markers, and clinical and neuropsychological assessment can be combined to measure the progression of mild cognitive impairment (MCI) and early Alzheimer's disease (AD). From the ADNI 1, ADNI 2, and ADNI GO phases, we selected a subset of 633 subjects with scans that had been noted with the “best” quality mark. As a result, the selected subset holds *N* = 1993 images with three class labels described above; *C* = 3. Besides, a random subset of 70% data was chosen for tuning and training stages, while the remaining 30% is for the test purpose. In addition, 629 images with a “partial” quality mark were selected in order to assess the performance under more complicated imaging conditions.  Table 1 briefly describes the demographic information for the ADNI selected cohort.

### 3.2. Processing of MRI Data

We used FreeSurfer, version 5.1 (a free available (http://surfer.nmr.mgh.harvard.edu/), widely used and extensively validated brain MRI analysis software package), to process the structural brain MRI scans and compute the morphological measurements [33]. FreeSurfer morphometric procedures have been demonstrated to show good test-retest reliability across scanner manufacturers and across field strengths [34]. The FreeSurfer pipeline is fully automatic and includes the next procedures: a watershed-based skull stripping [35], a transformation to the Talairach, an intensity normalization and bias field correction [36], tessellation of the gray/white matter boundary, topology correction [37], and a surface deformation [38]. Consequently, a representation of the cortical surface between white and gray matters, of the pial surface, and segmentation of white matter from the rest of the brain are obtained. FreeSurfer computes structure-specific volume, area, and thickness measurements. Cortical Volumes and Subcortical Volumes are normalized to each subject's Total Intracranial Volume (eTIV) [39].  Table 2 summarizes the five feature sets extracted for each subject, which are concatenated into the feature matrix **X** with dimensions *N* = 1993 and *D* = 324.

### 3.3. Tuning of ANN Model Parameter

**Figure 2:**
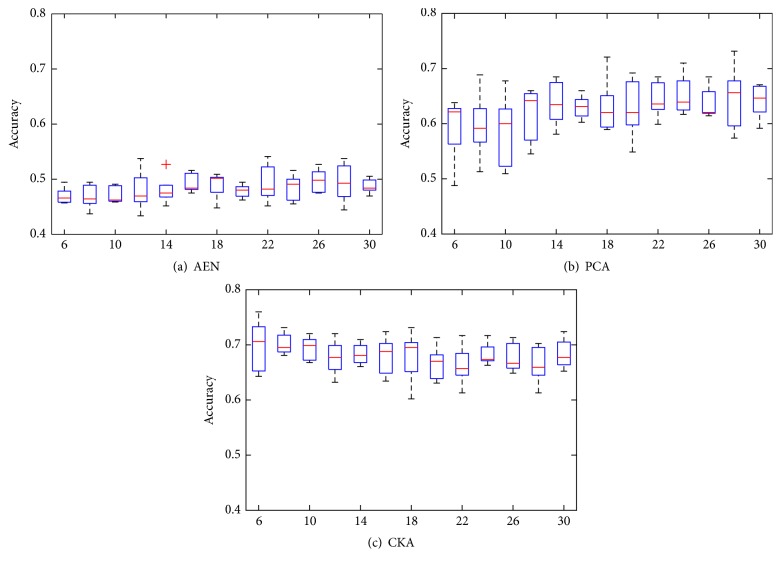
Artificial neural network performance along the number of nodes in the hidden layer (*m*_1_) for the three initialization approaches: autoencoder, PCA-based projection, and CKA-based projection. Results are computed under 5-fold cross-validation scheme.

Given input *D* = 324 MRI features for classification of the 3 neurological classes, we use the feedforward ANNs with one hidden layer: 324-input and 3-output neurons. An exhaustive search is carried out for tuning the single free parameter, namely, the number of neurons in the hidden layer (*m*_1_). We also compare our proposal against autoencoders (AEN) [20] and the well-known Principal Components Analysis (PCA) for the initialization stage. All of these approaches (AEN, PCA, and CKA) provide a projection matrix with an output dimension that, in this case, equates the hidden layer size. Thus, resulting projections are used as the initial weights for the first layer. Also, biases and output layer weights are randomly initialized. For a different number of neurons, Figure 2 shows the accuracy results obtained by each considered strategy of initialization using 5-fold cross-validation scheme. Since we look for the most accurate and stable network configuration, we chose the optimal net as the one with the highest mean-to-deviation ratio. The resulting search indicates that the best number of hidden neurons is accomplished at *m*_1_ = 20, *m*_1_ = 16, and *m*_1_ = 14 for AEN, PCA, and CKA approaches, respectively.

**Figure 3:**
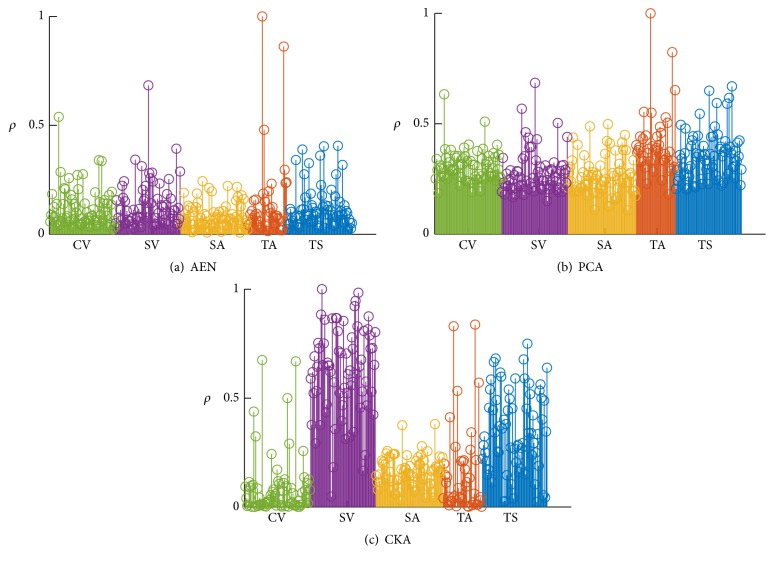
Relevance indexes grouped by feature type: Cortical Volume (CV), Subcortical Volume (SV), Surface Area (SA), Thickness Average (TA), and Thickness Std. (TS).

We further analyze the influence of each feature to the initialization process regarding the relevance criterion introduced in (11). Obtained results of relevance in Figure 3 show that the proposed CKA approach enhances the Subcortical Volume features at the time it diminishes the influence of most Cortical Volumes and Thickness Averages. The relevance of each feature set provided by AEN and PCA is practically the same. Hence, CKA allows the selection of relevant biomarkers from MRI.

### 3.4. Classifier Performance of Neurological Classes

As shown in Table 3, the ANN models that have been tuned for the three initialization strategies are contrasted with the best four performing approaches of the 2014 CADDementia challenge [17]. The compared algorithms are evaluated in terms of their classification performance, accuracy (*α*), area under the receiver-operating-characteristic curve (*β*), and class-wise true positive rate (*τ*_*p*_^*c*^) criteria, respectively, which are defined as

(12) α=∑ctpc+tnc∑cNc,τc=tpcNc,β=∑cβc·Nc∑cNc,

where *c* ∈ {NC, MCI, AD} indexes each class and *N* ^*c*^, *t*_*p*_^*c*^, and *t*_*n*_^*c*^ are the number of samples, true positives, and true negatives for the *c*th class, respectively. The area under the curve *β* is the weighted average of the area under the ROC curve of each class *β* ^*c*^. Presented results for the baseline approaches are the ones reported on the challenge for 354 images. Although the testing groups on the challenge and on this paper are not exactly the same, the amount of data, their characteristics, and the blind setup make those two groups equivalent for evaluation purposes.

**Figure 4:**
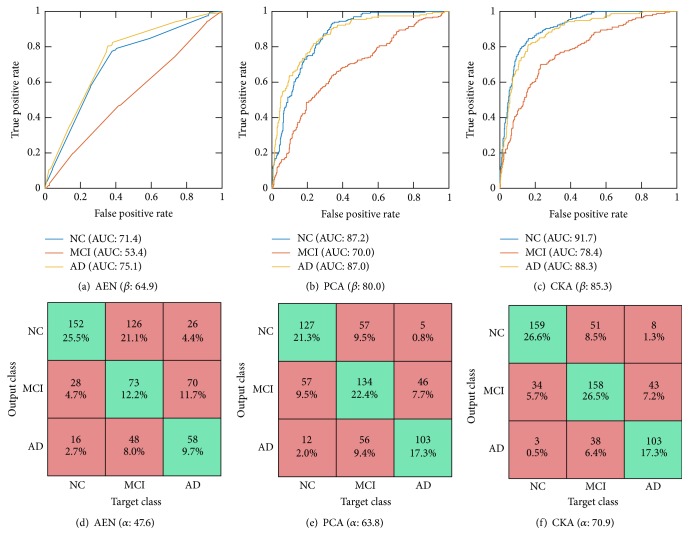
Receiver-operating-characteristic curve ((a), (b), and (c)) and confusion matrix ((d), (e), and (f)) on the 30% test data for AEN ((a) and (d)), PCA ((b) and (e)), and CKA ((c) and (f)) initialization approaches at the best parameter set of the ANN classifier.

As seen in Table 4 which compares the classification performance on the 30% “best” quality test set for considered algorithms, the proposed approach, besides outperforming other compared approaches of initialization, also performs better than other computer-aided diagnosis methods as a whole. For the “partial” quality images, as expected, the general performance diminishes in all ANN approaches. Nonetheless, the overall accuracy and AUC are still competitive with respect to the challenge winner. Based on the displayed ROC curves and confusion matrices for the ANN-based classifiers with the optimum parameter set (see Figure 4), we also infer that the proposed approach improves MCI discrimination.

## 4. Discussion

From the validation carried out above for MRI-based dementia diagnosis, the following aspects emerge as relevant for the developed proposal of ANN pretraining:

(i) As commonly implemented by the state-of-the-art ANN algorithms, the proposed initialization approach also has one free model parameter which is the number of hidden neurons. Tuning of this parameter is proposed to be carried out heuristically by an exhaustive search so as to reach the highest accuracy on a 5-fold cross-validation (see Figure 2). Thus, 24, 20, and 16 hidden neurons are selected for CKA, AEN, and PCA, respectively. As a result, the suggested CKA approach improves other pretraining ANN approaches (in about 10%) with the additional benefit of decreasing the performed parameter sensitivity.
(ii) We assess the influence of each MRI feature at the pretraining procedure regarding the relevance criterion introduced in (11). As follows from Figure 3, AEN and PCA ponder every feature evenly, restraining their ability to extract biomarkers. By contrast, CKA enhances the influence of Subcortical Volumes and Thickness Standard deviations at the time it diminishes the contribution of Cortical Volumes and Thickness Averages. Consequently, the proposed approach is also suitable for feature selection tasks.
(iii) In the interest of comparing, we contrast the developed ANN pretraining approach with the best four classification strategies of the 2014 CADDementia, devoted especially to dementia classification. From the obtained results, summarized in Table 4, it follows that proposed CKA outperforms other algorithms in most of the evaluation criteria and imaging conditions, providing the most balanced performance over all classes. Particularly for the 30% testing images, CKA increases by 7%-points the classification accuracy and average area under the ROC curve. It is worth noting that although Sørensen's approach accomplishes a *τ* ^NC^ value that is 18.5%-points higher than the proposal, its performance turns out to be biased towards the NC, yielding a worse value of MCI. That is, CKA carries out unbiased class performance of the dementia classification. In the case of “partial” quality images, in spite of the general performance reduction, CKA remains as the best ANN initialization approach. Moreover, the overall measures are still competitive with the results provided by the CADDementia challenge.
(iv) Figure 4 shows the per-class ROC curves and confusion matrices obtained by the contrasted approaches. In all cases, the area under the curve and accuracy for NC and AD classes are higher than the ones achieved by the MCI class (Figures 4(a)–4(c)). Hence, MCI classification from the incorporated MRI features remains a challenging task due to the following facts: the widely known MCI heterogeneity, the MCI being an intermediate class between healthy individuals and those diagnosed with Alzheimer's disease, and the possibility of MCI subjects eventually converting to AD or NC. Moreover, confusion matrices displayed in Figures 4(d)–4(f) confirm that NC and AD are suitable for distinction in most of the cases. Nevertheless, the MCI class introduces the most errors when considered as both target and output class. Therefore, particular studies on the mild cognitive impairment should improve the diagnosis [5, 40].

## 5. Conclusion and Future Work

In this paper, we propose a supervised method for initializing the training of artificial neural networks, aiming to improve the computer-aided diagnosis of dementia. Given a set of volume, area, surface, and thickness features extracted from the subject's brain MRI, the examined dementia diagnosis task consists of assigning subjects to the next neurological groups: normal control, mild cognitive impairment (MCI), or Alzheimer's disease. This dementia classification task is particularly challenging because MCI is a heterogeneous and intermediate category between NC and AD. Also, MCI subjects may convert to AD or come back to NC.

To improve the classification performance, we incorporate a matrix projecting the samples into a more discriminating feature space so that the affinity between projected features and class labels is maximized. Such a criterion is implemented by the centered kernel alignment (CKA) between the feature and target label kernels, providing two key benefits: (i) the only free parameter is the hidden dimension; (ii) a relevance analysis can be introduced to find biomarkers. As a result, our proposal of ANN pretraining outperforms the contrasted algorithms (7% of classification accuracy and area under the ROC curve) and reduces the class biasing, resulting in better MCI discrimination.

Nonetheless, the use of CKA implies a couple of restrictions. Firstly, the number of samples should be larger than input and output dimensions to avoid overfitted linear projections. We cope with this drawback by considering a large enough subset of samples for training purposes (about 1300). Secondly, attained projections must always be of lower dimension compared to the original feature space. In this case, the enhancement on class discrimination is due to the affinity between labels and features, not due to an increase of the dimension.

As future work, we plan to evaluate the CKA discriminative capabilities in other neuropathological tasks from MRI as predicting Alzheimer's conversion from MCI and attention deficit hyperactivity disorder classification. We also expect to develop a neural network training scheme using CKA as the cost function.

## Figures and Tables

**Table 1:** Demographic and clinical details of the selected ADNI cohort.

	“best” quality			“partial” quality		
	NC	MCI	AD	NC	MCI	AD
*N*	655	825	513	465	130	34
Age	74.9 ± 5.0	74.4 ± 7.4	74.0 ± 7.4	76.6 ± 6.4	76.0 ± 6.3	74.3 ± 6.5
Male	47.5%	39.5%	47.6%	70.1%	62.3%	70.6%
MMSE	29.0 ± 1.0	27.1 ± 2.5	21.9 ± 4.4	27.5 ± 2.0	21.2 ± 1.6	14.4 ± 2.8

**Table 2:** FreeSurfer extracted features. # stands for the number of features.

Type	# features	Units
Cortical Volumes (CV)	70	mm^3^
Subcortical Volumes (SV)	42	mm^3^
Surface Area (SA)	72	mm^2^
Thickness Average (TA)	70	mm
Thickness Std. (TS)	70	mm

**Total**	324	

**Table 3:** Best performing algorithms in the 2014 CADDementia challenge [17].

Algorithm	Features	Classifier
Abdulkadir	Voxel-based morphometry	Support Vector Machine
Ledig	Volume and intensity relations	Random Forest classifier
Sørensen	Volume, thickness, shape, and intensity relations	Regularized Linear Discriminant Analysis
Wachinger	Volume, thickness, and shape	Generalized Linear Model

**Table 4:** Classification performance on the testing groups for considered algorithms under evaluation criteria. Top: baseline approaches. Bottom: ANN pretrainings.

Algorithm	*α*	*τ* ^NC^	*τ* ^MCI^	*τ* ^AD^	*β*	*β* ^NC^	*β* ^MCI^	*β* ^AD^
	2014 CADDementia							
Sørensen	**63.0 **	** 96.9 **	28.7	** 61.2 **	** 78.8 **	86.3	** 63.1 **	87.5
Wachinger	59.0	72.1	51.6	51.5	77.0	83.3	59.4	**88.2 **
Ledig	57.9	89.1	41.0	38.8	76.7	**86.6 **	59.7	84.9
Abdulkadir	53.7	45.7	**65.6 **	49.5	77.7	85.6	59.9	86.7

	“best” quality testing							
NN-AEN	47.6	73.4	33.1	38.1	64.9	71.4	53.4	75.1
NN-PCA	63.8	70.4	56.7	66.9	80.0	87.2	70.0	87.0
NN-CKA	**70.9 **	78.4	** 66.6 **	** 68.3 **	** 85.3 **	** 91.7 **	** 78.4 **	** 88.3 **

	“partial” quality							
NN-AEN	62.9	64.6	46.4	32.0	77.0	82.5	65.6	72.5
NN-PCA	64.4	67.6	**49.3 **	26.0	78.4	82.3	67.5	79.2
NN-CKA	**65.2 **	68.6	38.6	42.0	** 81.6 **	85.7	** 70.1 **	82.4

## Appendix

### Gradient Descend-Based Optimization of CKA Approach

The explicit objective function of the empirical CKA in (9) yields [32]

(A.1) ρ^CKAKW,B=log⁡trKWHBH−12log⁡trKWHKWH+ρ0,

with *ρ*_0_ ∈ *ℝ* being a constant independent of **W**. We then consider the gradient descent approach to iteratively solve the optimization problem. To this end, we compute the gradient of the explicit function in (A.1) with respect to **W** as

(A.2) ∇Wρ^CKAKW,B=−4WG∘KW−diag⁡1⊤G∘KWXW⊤,

where diag(·) and ∘ denote the diagonal operator and the Hadamard product, respectively. **G** ∈ *ℝ* ^*N*×*N*^ is the gradient of the objective function with respect to the kernel matrix **K**_**W**_:

(A.3) G∇KWρ^CKAKA,B=HBHtrKWHBH−HKWHtrKWHKWH.

As a result, the updating rule for **W**, given the initial guess **W** ^0^, becomes

(A.4) $$\begin{array}{c} W^{t+1}=W^{t}-\mu_{W}^{t}\nabla_{W^{t}}\left(\;{\hat{\rho }}_{CKA}\left(\;K_{W},B\;\right)\;\right), \end{array}$$

with *μ*_*W*_^*t*^ ∈ *ℝ* ^+^ being the step size of the updating rule and **W** ^*t*^ being the estimated projection matrix at iteration *t*.
